# Increased m6A-RNA methylation and FTO suppression is associated with myocardial inflammation and dysfunction during endotoxemia in mice

**DOI:** 10.1007/s11010-021-04267-2

**Published:** 2021-09-28

**Authors:** Praveen K Dubey, Mallikarjun Patil, Sarojini Singh, Shubham Dubey, Paras Ahuja, Suresh Kumar Verma, Prasanna Krishnamurthy

**Affiliations:** 1 Department of Biomedical Engineering, Schools of Medicine and Engineering, The University of Alabama at Birmingham, AL 35294, USA; 2 Science and Technology Honors College, The University of Alabama at Birmingham, AL 35294, USA; 3 Division of Cardiovascular Disease, School of Medicine, The University of Alabama at Birmingham, AL 35294, USA

**Keywords:** Endotoxemia, Cardiac dysfunction, RNA methylation, Sepsis, FTO

## Abstract

Endotoxemia triggers life-threatening immune and cardiovascular response that leads to tissue damage, multi-organ failure, and death. The understanding of underlying molecular mechanisms is still evolving. N6-methyladenosine (m6A) RNA modification plays key regulatory role in numerous biological processes. However, it remains unclear whether endotoxemia alters RNA methylation in the myocardium. In the current study, we investigated the effect of LPS-induced endotoxemia on m6A-RNA methylation and its implications on myocardial inflammation and left ventricular (LV) function. Following LPS administration, mice showed increases in m6A-RNA methylation in the myocardium with a corresponding decrease in the expression of Fat mass and obesity-associated protein (FTO, an m6A eraser/demethylase). The changes were associated with a significant increase in expression of myocardial inflammatory cytokine genes, such as IL-6, TNF-α, IL-1β, and reduced LV function. Moreover, rat cardiomyoblasts (H9c2) exposed to LPS showed similar changes (with increase in m6A-RNA methylation and inflammatory cytokine genes, whereas downregulation of FTO). Furthermore, methylated RNA immunoprecipitation (MeRIP) assay showed hypermethylation and increase in the expression of IL-6 and TNF-α genes in LPS-treated H9c2 cells as compared to untreated cells. Interestingly, FTO knockdown in cardiomyocytes mimicked the above effects. Taken together, these data suggest that endotoxemia-induced m6A methylation might play a critical role in expression of cardiac proinflammatory cytokines, and modulation of m6A methylation might limit myocardial inflammation and dysfunction during endotoxemia.

## Introduction

Sepsis, dysregulated immune response to infection, is a serious life-threatening condition that affects more than 30 million people worldwide, with an estimated 1.7 million adult cases annually in the United States [1, 2]. Endotoxemia, caused by circulating bacterial lipopolysaccharide (LPS), is implicated in the pathogenesis of sepsis and septic shock [3]. If left untreated at an early stage, sepsis can lead to septic shock, cardiac injury, multiple organ failure, and the Endotoxemia induces myocardial contractile dysfunction through upregulation of myocardial inflammatory responses, which leads to cardiac cell death, calcium imbalance, autophagy, microvascular dysfunction, and mitochondrial damage [1, 4–6]. However, the underlying molecular mechanisms are still elusive.

N6-methyladenosine (m6A) RNA-modification is one of the important posttranscriptional modifications of eukaryotic mRNA that plays a regulatory role in diverse biological processes [7, 8]. m6A modification is dynamically regulated by a set of enzymes, known as writers (methyltransferases), erasers (demethylases), and readers (proteins that recognize and interact with the m6A site [9]. Alteration in these enzymes can affect the degree of m6A modification and subsequent splicing, stability, translation, and subcellular localization of their target mRNAs [9–11]. These changes could result in aberrant gene expression in multiple pathophysiological processes [7, 12]. Fat mass-and obesity-associated (FTO) protein is a nucleic acid demethylase (removes the methyl group from RNA) and has been reported to regulate the progression of various cardiac diseases, such as hypertrophic cardiomyopathy, interventricular septal and atrioventricular defects, arrhythmias, and coronary heart diseases [13–15]. Previous studies have shown downregulation of FTO in the myocardium of failing mammalian hearts with a concomitant decrease in cardiomyocyte contractility [14]. Furthermore, alterations in m6A RNA methylation have been implicated in the development of heart failure [11, 14, 15]. However, despite its functional importance in various fundamental biological processes, the role of FTO-dependent m6A RNA modification in the myocardium during endotoxemia and its implications is still unknown.

In the present study, we investigated the influence of LPS-induced endotoxemia on cardiac m6A-RNA modification and FTO expression. We also investigated if altered FTO expression in cardiomyocytes could affect the m6A modification of inflammatory cytokines. We demonstrated that LPS-induced endotoxemia increases global m6A-RNA methylation in the mouse myocardium with a significant downregulation of FTO and LV functions. Further, reduced FTO activity resulted in increased m6A methylation of IL-6, IL-1β, and TNF-α transcripts in cardiomyocytes.

## Materials and methods

### Vertebrate animals

All animal experiment protocols were approved by the University of Alabama at Birmingham Institutional Animal Care and Use Committee (IACUC). C57BL/6J mice (10-12-weeks-old, Jackson Laboratory; Bar Harbor, ME) were allowed to acclimatize for 10 days in a pathogen-free animal facility with a standard rodent diet and water. Mice were randomly assigned into two experimental groups: i) the control group (n = 5), intraperitoneally administered 300 μl of 0.9% saline (vehicle) and ii) LPS group (n=5), intraperitoneal administration of 15 mg/kg body weight of LPS (*Escherichia coli* serotype 0111:B4, Sigma Aldrich, USA) in 300 μl of 0.9% saline. After 6 hours, Echocardiography was recorded and mice were euthanized, hearts were removed, and quick-frozen in liquid nitrogen. Tissues were stored at −80°C for further analysis.

### Echocardiography and LV function assessment

Echocardiograms of all animals in each group were recorded pre- and post-LPS injection (6 hours) using a Vevo 2100 imaging system (VisualSonics, Toronto, Canada). Mice were anesthetized with 3% isoflurane and maintained at 1% isoflurane during echocardiography. Heart rate and body temperature were monitored with ECG tracing and a rectal thermometer, respectively. Left ventricular fractional shortening (FS) and ejection fraction (EF) were calculated to assess cardiac function. LV posterior wall thickness at end-diastole and end-systole (LVPW; d and LVPW; s), left ventricular internal diameter at end-diastole and end-systole (LVID; d and LVID; s), and left ventricular volume at end-diastole and end-systole (LV Vol; d and LV Vol; s) were obtained from the m-mode tracings.

### Cell culture and treatment

Rat cardiomyoblast H9c2(2-1) (cat# CRL-1446) was purchased from ATCC (American Type Culture Collection, USA) and cultured in Dulbecco’s Modified Eagle’s Medium (DMEM, Life Technologies, Grand Island, NY) supplemented with 10% fetal bovine serum (FBS, ATCC, Manassas, VA) and 1% Penicillin-Streptomycin (Life Technologies) in a humidified incubator at 37°C with 5% CO_2_. Cells were plated onto 6-well, 12-well, or 10-cm culture dishes with or without LPS (1 μg/ml) for 6 hours.

### RNA Interference

H9c2 cells were seeded into tissue culture plates. After reaching 70-80% confluency, the cells were transfected either with rat FTO siRNA (cat# D-093125-02-0005, siGENOME, PerkinElmer, USA) or scramble control (Cat # 4390843 Silencer™ Select Negative Control#1, ThermoFisher Scientific) using Lipofectamine RNAiMAX reagent (cat # 13778075, Life Technologies, USA) following the manufacturer’s instruction. After 48 hours of transfection, cells were treated with or without LPS (1 μg/ml) for 6 hours.

### Dot blot analysis of N6-methyladenosine (mM6A) RNA modification

Total RNA and polyA+ RNA was isolated from the cardiac tissue of vehicle- or LPS-administered mice or H9c2 cells treated with LPS using RNeasy mini kit (for total RNA; cat# 74106, Qiagen) and Dynabeads® mRNA purification kit (cat# 61006, Ambion), according to the manufacturers’ instructions. RNA was quantified using Nanodrop and equal amounts of RNA were crosslinked onto Hybond-N discs (Amersham Hybond-N membranes, cat# RPN82N; Cytiva) using a UV crosslinker (1200 mJoule for 1min; Spectroliner, cat# XL-1500). Negative and positive controls for m6A (Epigentek Group Inc, cat# P9005-96) were also included on the dot blots. The membrane was quickly washed and blocked using 5% non-fat dry milk in 0.1% phosphate-buffered saline with Tween-20 (PBST) supplemented with RNaseOUT™ (cat#, 10777019, ThermoFisher Scientific). The membrane was incubated overnight at 4 °C with rabbit anti-m6A antibody (1:500, cat# A-1802-100, EpiGenTek, USA), followed by incubation with HRP-conjugated secondary anti-rabbit antibody. The membrane was washed and visualized by an enhanced chemiluminescence (Pierce) detection system. Images were acquired using a ChemiDoc™ Touch Imaging System (Bio-Rad, USA). Finally, the membrane was stained with Methylene blue for loading control. The signal intensity of the dot blots was analyzed using NIH-ImageJ software.

### Protein extraction and Western blot analysis

Protein lysates were prepared using RIPA Cell Lysis Buffer (cat# J63324, Alfa Aesar) supplemented with phosphatase inhibitor (cat# P5726, Millipore Sigma) and Halt™ protease inhibitor (cat# 87786, ThermoFisher Scientific) cocktails according to the manufacturers’ instructions. Equal amounts of proteins were separated on 4-12% Mini-PROTEAN TGX stain-free protein gels (cat# 4568093, Bio-Rad Laboratories) and transferred to polyvinylidene difluoride (PVDF) membranes using Trans-Blot Turbo transfer system (Bio-Rad Laboratories). The blots were incubated overnight at 4°C with primary antibodies against FTO (cat# 27226-1-AP, Proteintech), N6-methyladenosinem6A (cat# A-1801-100, EpiGenTek), Lamin B1 (cat# 12987-1-AP, Proteintech), GAPDH (cat# 60004-1-Ig, Proteintech), and β-Tubulin (cat# 10094-1-AP, Proteintech). The blots were washed and then incubated with HRP-conjugated secondary antibodies against mouse (cat# SA00001-1, Proteintech) and rabbit (cat# SA00001-2, Proteintech) at room temperature for 1 hour according to the manufacturer’s instructions. The blots were developed by an enhanced chemiluminescence (Pierce) detection system, and images were acquired using a ChemiDoc™ Touch Imaging System (Bio-Rad, USA). Densitometric analyses were performed using ImageJ (NIH) software.

### Subcellular fractionation

H9c2 rat cardiomyoblasts were treated with or without LPS for 6 hours. Cytoplasmic and nuclear proteins were extracted using NE-PER™ Nuclear and Cytoplasmic Extraction Reagents (cat# 78835, ThermoFisher Scientific) following the manufacturer’s instructions. Protease and phosphatase inhibitor cocktails were added to the extraction reagents before use. Fractionated proteins were resolved on denaturing SDS-PAGE gels, and purity of the fractions was confirmed by immunoblotting with antibodies against GAPDH (cytoplasmic marker) and Lamin B1 (Nuclear marker).

### RNA isolation and quantitative qRT-PCR

Total RNA from mice heart tissue and H9c2 cells was extracted using a Qiagen RNA extraction kit (cat# 74106, Qiagen) according to the manufacturer’s instructions. The RNA was reverse transcribed using RevertAid First Strand cDNA Synthesis Kit (cat# K1691, ThermoFisher Scientific). Quantitative real-time PCR (qPCR) was performed with gene-specific primers (Supplementary Table 1) in a QuantStudio 3 system (Applied Biosystems, ThermoFisher Scientific) using the PowerUp™ SYBR™ Green Master Mix (cat# A25778, ThermoFisher Scientific) according to the manufacturer’s instructions. Expression of the target genes was normalized to housekeeping genes (GAPDH, β-actin, or 18S rRNA). Gene expression was represented as fold change versus control.

### M6A-RNA immunoprecipitation (MeRIP assay).

To assess the relative abundance of methylated RNA in the cells, MeRIP assay was performed using a RIP-Assay Kit (to pulldown RNA-protein complex, cat# RN1001, MBL) along with N6-methyladenosine antibody (cat# A-1801-100, EpiGenTekM6A) according to the manufacturers’ instructions. After pulldown, the RNA quality and quantity were measured using a microplate spectrophotometer (Epoch 2, BioTek, USA). The RNA was reverse transcribed, and qPCR was performed as described above. Cycle threshold (Ct) values were used to determine the relative enrichment of mRNA.

### Cell proliferation assay

Cell proliferation was assessed with a CyQUANT Cell Proliferation Assay (cat# c7026, ThermoFisher Scientific) as per the manufacturer’s instructions. H9c2 cells were seeded onto 96-well plates and incubated overnight. After stimulation with LPS (24 hours), the cells were incubated with CyQuant GR dye in a cell-lysis buffer. The absorbance was detected on a microplate spectrophotometer (Epoch 2, BioTek, USA) with an excitation maximum of 480 nm and an emission maximum of 520 nm.

### Statistical analyses

The data were analyzed using two tailed unpaired t-Test for comparison of two groups or one-way analysis of variance (ANOVA) following Brown-Forsythe test and Bartlett’s statistic (corrected). All the values are presented as mean ± SEM. The probability (P) values of ≤0.05 were considered statistically significant.

## Results

### Increased m6A-RNA methylation in the myocardium of endotoxemic mouse.

**Figure 1.**
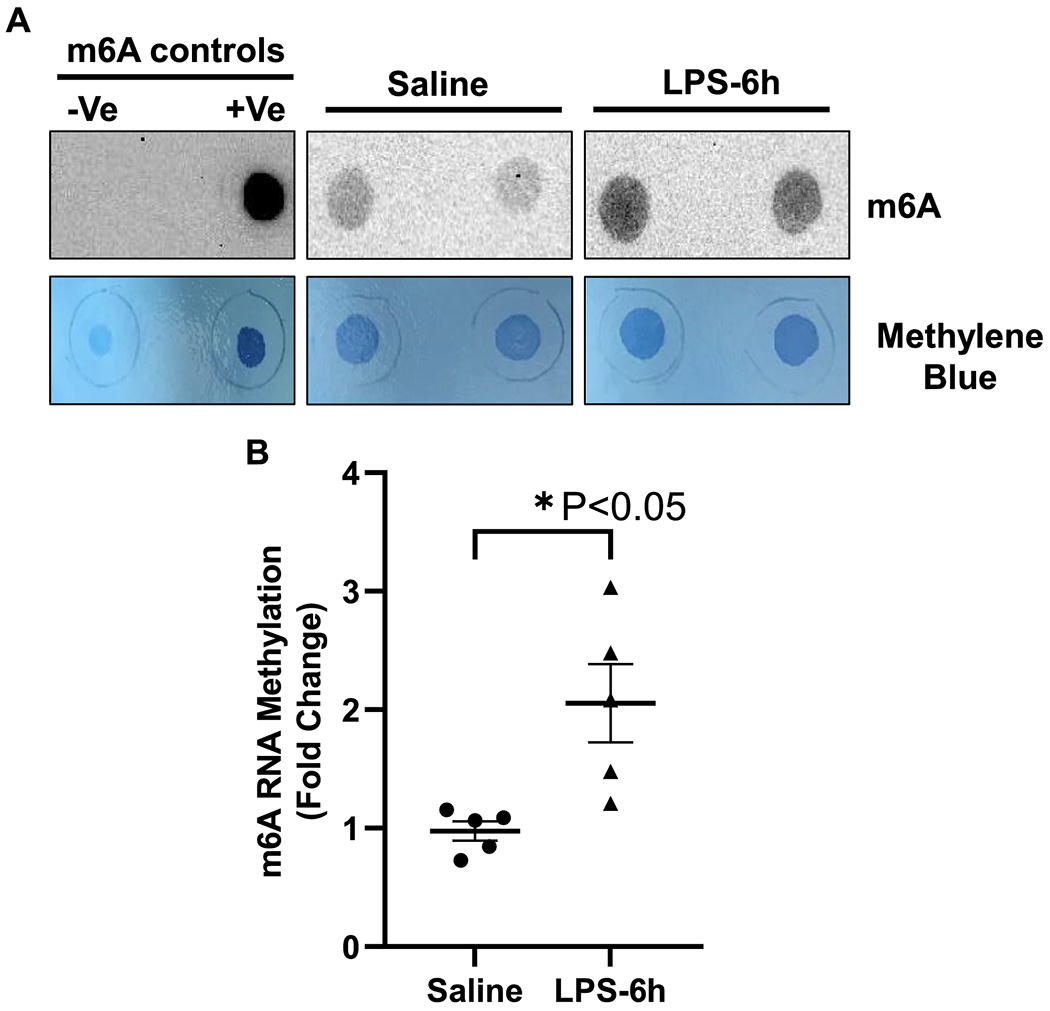
LPS induced endotoxemia upregulate RNA methylation (m6A) in cardiac tissue. (A) Dot Blot of polyadenylated RNA (poly A+) isolated from saline- and LPS-injected mice heart and immunoblotted with m6A antibody along with control samples (+ve, m6A-positive RNA; and −ve, non-m6A RNA). Methylene blue staining was used as a loading control. (B) Densitometry of m6A methylation Dot Blot in LPS- and saline-injected mice (n=5). All data are represented as means SEMs. Data were analyzed using two tailed unpaired t test.

RNA modifications, such as methylation of N6-methyladenosine (m6A), are important posttranscriptional regulatory mechanisms in diverse biological processes [11, 14, 16, 17]. To determine the effect of endotoxemia on m6A-RNA methylation in the heart, polyadenylated RNA was extracted from the cardiac tissue of the vehicle or LPS-administered mice, and RNA dot blot was performed using an anti-m6A antibody. The RNA dot blot analysis showed a significant increase in global m6A-RNA methylation in the myocardium of LPS-treated mice as compared to the vehicle group (~2-fold LPS vs. vehicle, P=0.0132, Fig. 1A and 1B). Methylene blue staining was used as a loading control. Negative and positive controls for m6A were also included on all membranes.

### Endotoxemia inhibited myocardial expression of fat mass- and obesity-associated protein (FTO), an m6A eraser/demethylase.

**Figure 2.**
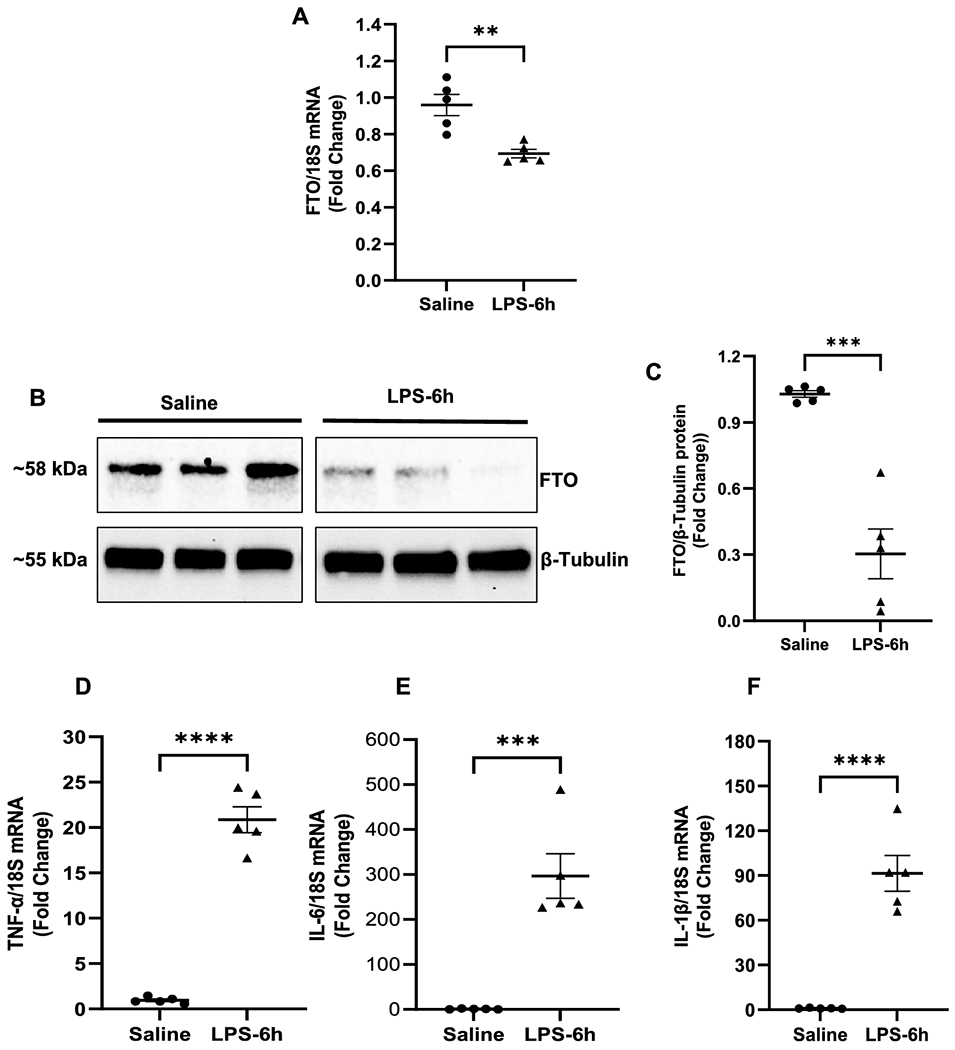
LPS-induced endotoxemia downregulates expression of FTO in mice Heart. (A) Relative expression of FTO mRNA normalized to 18S rRNA in the heart. (B) Representative immunoblot showing the expression of FTO and β-Tubulin in the cardiac tissues from LPS- and saline- injected mice. (C) Densitometric quantification of FTO protein after normalization with β-Tubulin. (D, E, F) Quantification of proinflammatory cytokines IL-6, TNF-α and IL-1β mRNA in myocardium (data normalized to 18S rRNA). Values are shown as fold change compared to the saline group. All data are represented as means SEMs. Data were analyzed using two tailed unpaired t-test.

m6A modification is a dynamic process and is tightly regulated by methyltransferases, demethylases, and readers. Alteration in these enzymes could change m6A modification and subsequent mRNA splicing, stability, and translation [15, 17–19]. Previous reports have shown that FTO, an m6A demethylase, regulates cardiac function in the heart pathophysiology [13–15]. To determine whether increased m6A-RNA methylation in the endotoxemic mouse heart is associated with changes in demethylase activity of FTO, we analyzed the myocardial expression of FTO in the saline- and LPS-administered mice. Interestingly, we observed that myocardial expression of FTO was significantly downregulated both at mRNA (Fig. 2A) and protein level (Fig. 2B and 2C) in LPS-treated mice as compared to the saline group.

### m6A-RNA increase was associated with upregulation of myocardial inflammatory markers and LV dysfunction in endotoxemic mice.

Cardiovascular dysfunction is one of the important consequences of endotoxemia or septic shock and contributes to high morbidity and mortality [1, 5]. Cytokine storm is a major cause of organ failure in patients with septic shock [20]. To determine the effect of endotoxemia on myocardial inflammation, proinflammatory cytokines genes (IL-6, TNF-α, and IL-1β) were measured in the saline and LPS-administered mice hearts. LPS-administration resulted in a robust increase in mRNA expression of IL-6 (~300-fold, P<0.0001, Fig. 2D), TNF-α (~21-fold, P<0.0001, Fig. 2E), and IL-1β (~90-fold, P<0.0001, Fig. 2F) in the myocardium of LPS-treated mice as compared to the saline group.

**Figure 3.**
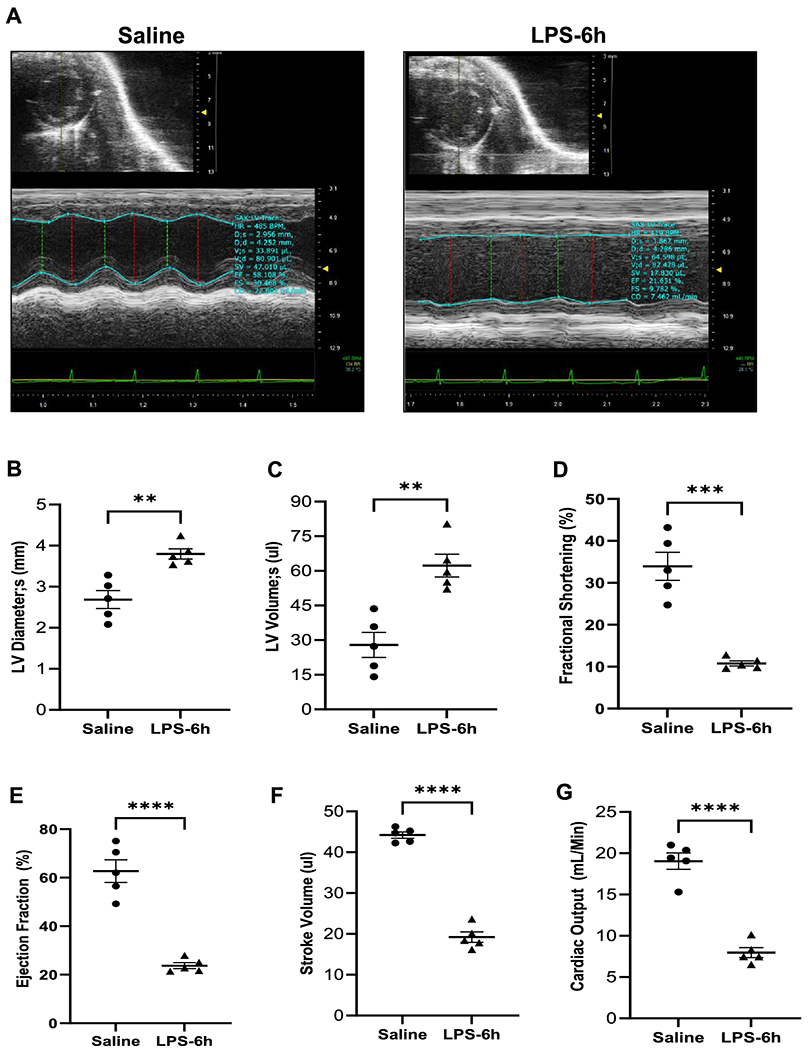
LPS-mediated endotoxemia induced cardiac dysfunction and inflammation. A. Representative M-mode echocardiographic images of the saline- and LPS-treated mice. Echocardiographic quantification of (B) Left ventricle internal diameter at systole (LVIDs), left ventricular systolic volume (C), Fractional shortening (%FS) (D), Ejection fraction (% LVEF) (E), stroke volume (F), and cardiac output (G). Data are represented as mean±SEM (n=5 for each group). Statistical analysis was done by two tailed unpaired t-test and * P<0.05, ** P<0.01, *** P<0.001 ****P<0.0001.

Further, left ventricular (LV) functional and structural parameters were assessed by echocardiography. M-mode echocardiography tracing (Fig. 3A) and data analyses (Fig. 3B-G) showed a significant change in LV parameters in LPS-administered mice compared to the saline group, endotoxemic (LPS) mice showed a significant increase in LV chamber diameter at systole (LVID; s: 2.68±0.22 mm saline group vs. 3.79±0.12 mm LPS, P=0.0022, Fig. 3B) and LV volume at systole (27.95±5.38 μl saline group vs. 62.30±4.97 μl LPS, P=0.0016, Fig. 3C). A corresponding significant reduction was observed in percent fractional shortening (%FS, 33.93±3.34% saline group vs. 10.79±0.59% LPS, P=0.0001, Fig. 3D), percent LV ejection fraction (%EF, 62.68±4.67% saline group vs. 23.72±1.22% LPS, P<0.0001, Fig. 3E), stroke volume (44.21±0.76 μl saline group vs. 19.22±1.47 μl LPS, P<0.0001, Fig. 1F), and cardiac output (19.04±0.9912 ml/min saline group vs. 7.96±0.61 ml/min LPS, P<0.0001, Fig. 1G) 6 hours post-LPS administration compared to the saline group. Heart rate (430±18.20 BPM saline vs. 413±4.72 BPM LPS, P=0.3989, Supplementary Fig. S1A), LV volume at diastole (72.16±5.42 μl saline group vs. 81.52±5.87 μl LPS, P=0.2751, Supplementary Fig. S1B), and LV chamber diameter at diastole (LVID; d: 4.04±0.13 mm saline group vs. 4.25±0.18 mm LPS, P=0.2635, Supplementary Fig. S1C) were comparable between the two groups.

### Increased m6A-RNA methylation and associated upregulation of inflammatory cytokines in the LPS-treated cardiomyocytes.

**Figure 4.**
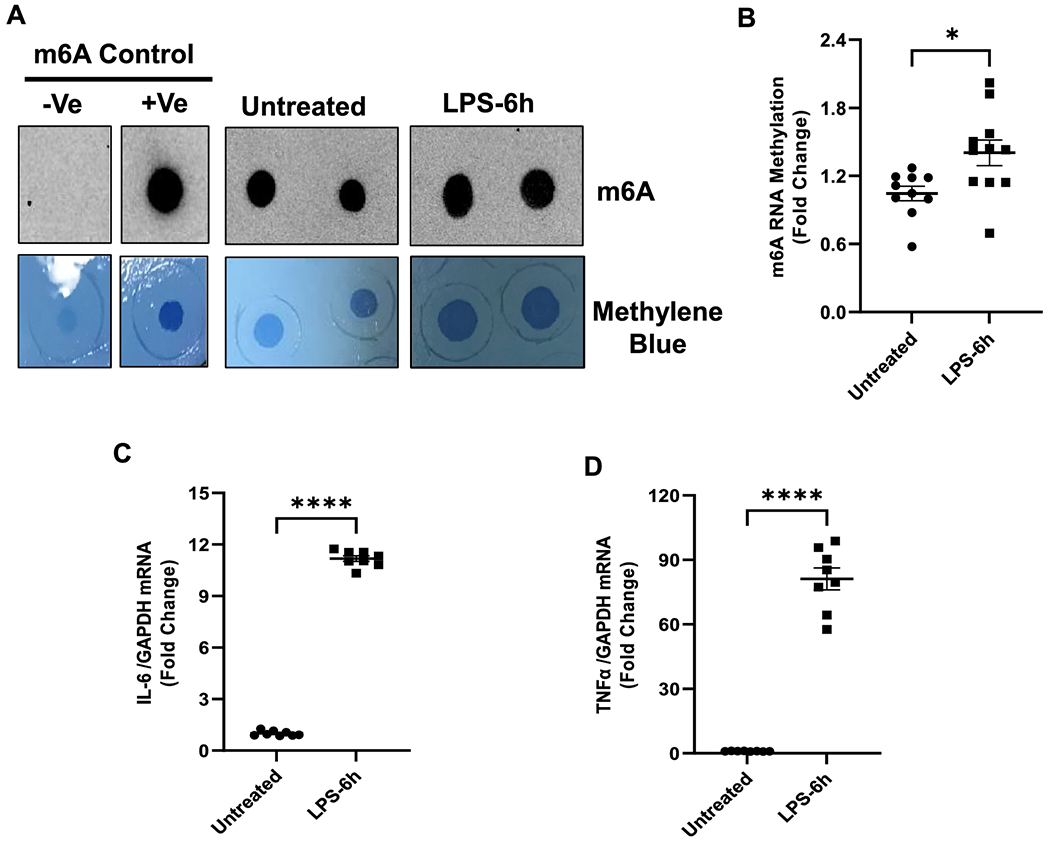
LPS induced RNA methylation (m6A) in Rat Cardiomyocytes (H9C2 cells). (A) Dot Blot analysis of poly(A)+ RNA isolated from H9C2 cells treated with or without LPS for 6 hrs, along with control samples (+ve, m6A-positive RNA; and −ve, non-m6A RNA). Methylene blue staining was used as a loading control. (B) Densitometry of m6A methylation in LPS-treated and untreated control cells normalized to methylene blue. (C, D) Proinflammatory cytokines (IL-6 and TNF-α) measured by qPCR, normalized to GAPDH, n=6–10 per group All data are represented as means SEMs. Data were analyzed using two tailed unpaired t test. * P<.05, ****P<0.0001

In our animal model of endotoxemia, we found increased global m6A-RNA methylation in the myocardium of LPS-administered mice compared to the saline-administered group. To further validate our findings, rat cardiomyoblasts (H9c2) were treated with or without LPS (1μg/ml) for 6 hours, and RNA dot-blot analysis was performed on the extracted polyadenylated mRNA. The mRNA expression of inflammatory cytokines was also evaluated by qRT-PCR. Similar to the *in vivo* findings, we observed an increase in global m6A methylation in the LPS-treated cells compared to the untreated control cells (Fig. 4A and 4B). Furthermore, we also observed a corresponding increase in the relative mRNA expression of proinflammatory cytokines IL-6 and TNF-α ( Fig. 4C and 4D).

### LPS stimulation altered the expression and subcellular localization of FTO in rat cardiomyocytes.

**Figure 5.**
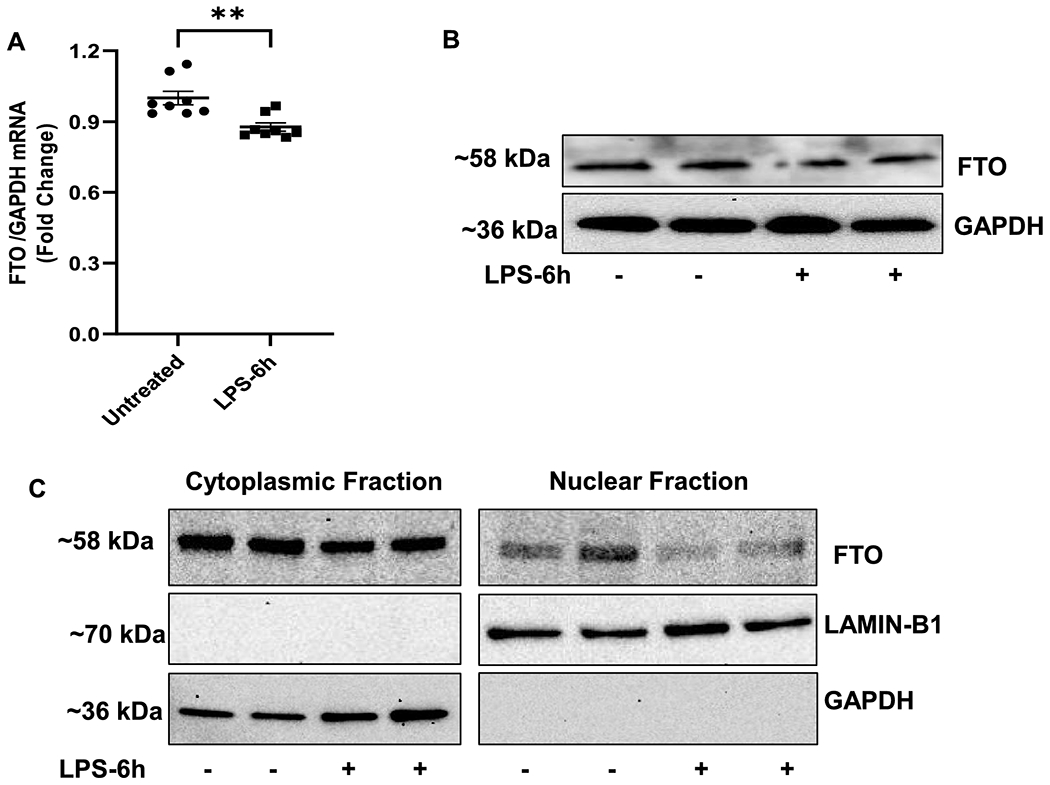
Expression and localization of FTO in rat cardiomyocyte cell (H9c2) after LPS treatment. (A) Relative expression of FTO mRNA in LPS treated cells as compared to untreated after normalization to GAPDH (n=8). All data are represented as means SEMs. Data were analyzed using two tailed unpaired t-test. (B) Representative immunoblot of FTO in LPS and untreated control H9C2 cells (C) Localization of FTO in cytosolic and fraction from LPS treated and untreated H9c2 cells. LaminB1 and GAPDH used as nuclear and cytoplasmic markers. ** P<0.01.

We stimulated H9c2 cells with LPS (1μg/ml) for 6 hours, and mRNA and protein expression of FTO was assessed. Similar to our *in vivo* observation, the mRNA and protein expression of FTO was downregulated in the LPS-treated cells as compared to the untreated cells (Fig. 5A and 5B).

Next, we analyzed whether LPS stimulation alters the subcellular localization of FTO. The expression of FTO was analyzed by western blotting in nuclear and cytoplasmic fractions of the H9c2 cells treated with or without LPS. Interestingly, we observed that the protein expression of FTO was downregulated in both cytoplasmic as well as in nuclear fraction (Fig. 5C).

### FTO knockdown in rat cardiomyocytes upregulated m6A-RNA methylation and expression of proinflammatory cytokines.

**Figure 6.**
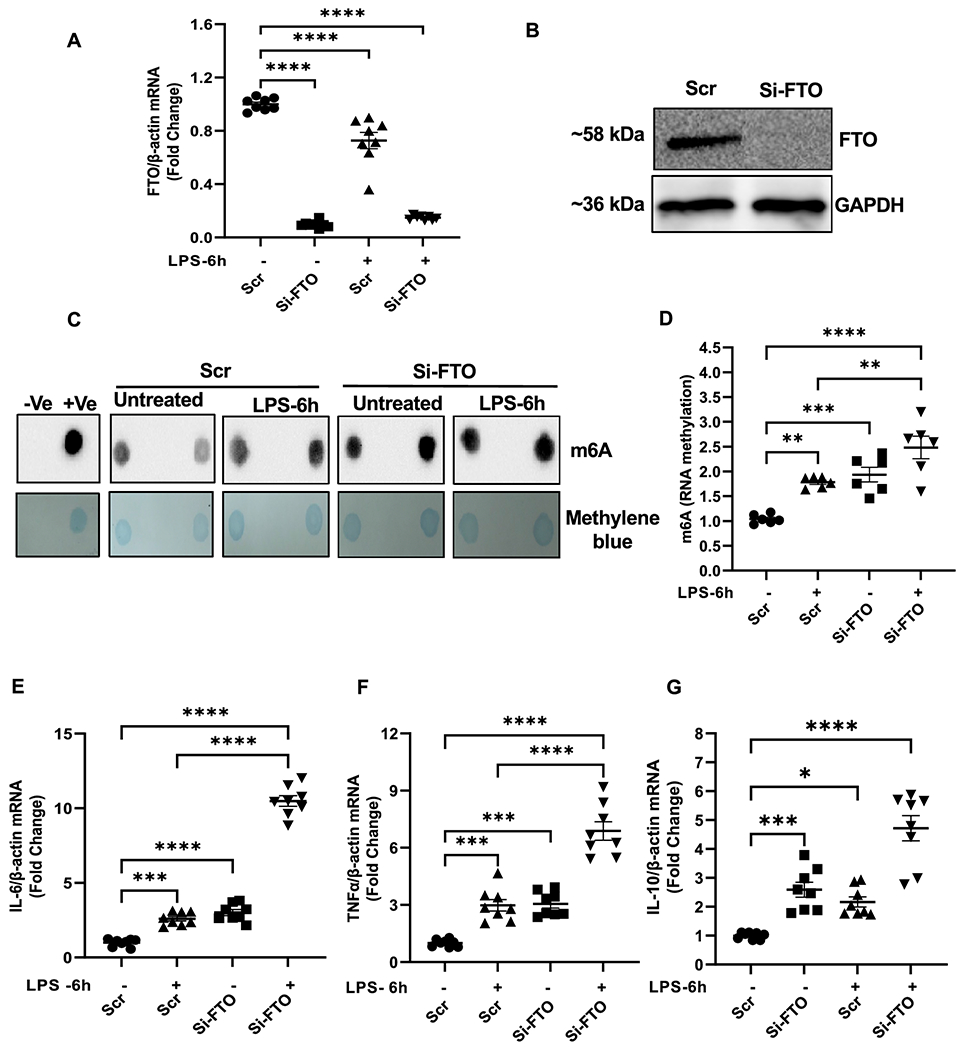
FTO knockdown in rat cardiomyocyte (H9C2) cells upregulates m6A-RNA methylation and proinflammatory cytokines (TNFα and IL6). (A, B) mRNA and protein Knock-down efficiency of FTO in H9C2 cells after 48hrs of transfection with FTO- and Scr- (Scramble) siRNA. (C) Dot Blot analysis of total RNA isolated from Scr (Scramble) control and FTO knock-down H9C2 cells treated with or without LPS. Control samples are included in the blot (+ve, m6A-positive RNA; and −ve, non-m6A RNA). Methylene blue staining was used as a loading control. (D) Densitometric analysis of m6A fold change in Scr (Scramble) and FTO knock down cells after LPS treated cells. (E, F) Relative expression IL-6 and TNF-α mRNA fold change in FTO knockdown cells as compared to scr (Scramble) control cells treated with and without LPS (1 ug/ml) for 6 hrs measured by qPCR (n=8). Data were normalized with housekeeping gene (β-actin) and represented as fold change compared to untreated cells. All data are represented as means SEMs. Data were analyzed using one-way ANOVA following Bartlett’s statistic (corrected) method to compare each groups. *** P<0.001 ****P<0.0001

Cytokine storm plays a major role in organ failure during endotoxemia and septic shock [1, 5, 20]. We found that the downregulation of FTO in the myocardium of endotoxemic mice was accompanied by a robust surge in inflammatory cytokines. To further strengthen our findings, we performed RNA interference studies. H9c2 cells were transfected either with FTO-siRNA or scrambled control-siRNA for 48 hours and then stimulated with LPS for 6 hours. Knockdown efficiency of FTO was confirmed by qRT-PCR (Fig. 6A) and immunoblotting (Fig. 6B). The RNA dot blot analysis showed that m6A methylation was elevated in the FTO knockdown cells both at the basal level and after LPS stimulation (Fig. 6C and 6D). Interestingly, knockdown of FTO also upregulated IL-6, TNF-α and IL-10 expression (Fig. 6E, 6F, and 6G), confirming that FTO-induced increased m6A methylation could cause an inflammatory surge. However, we did not notice a change in expression of IL-1β in any of the groups (Supplementary Figure S2).

To rule out the possibilities that increased methylation of RNA is due to increased cell number or m6A methylation induces cell proliferation, we assessed cell proliferation in the FTO-silenced H9c2 cells with or without LPS stimulation for 24 hours. We did not observe any significant changes in cell proliferation among the groups (Supplementary Fig. S3).

### Downregulation of FTO caused hypermethylation of IL-6 and TNF-α mRNA transcripts.

**Figure 7.**
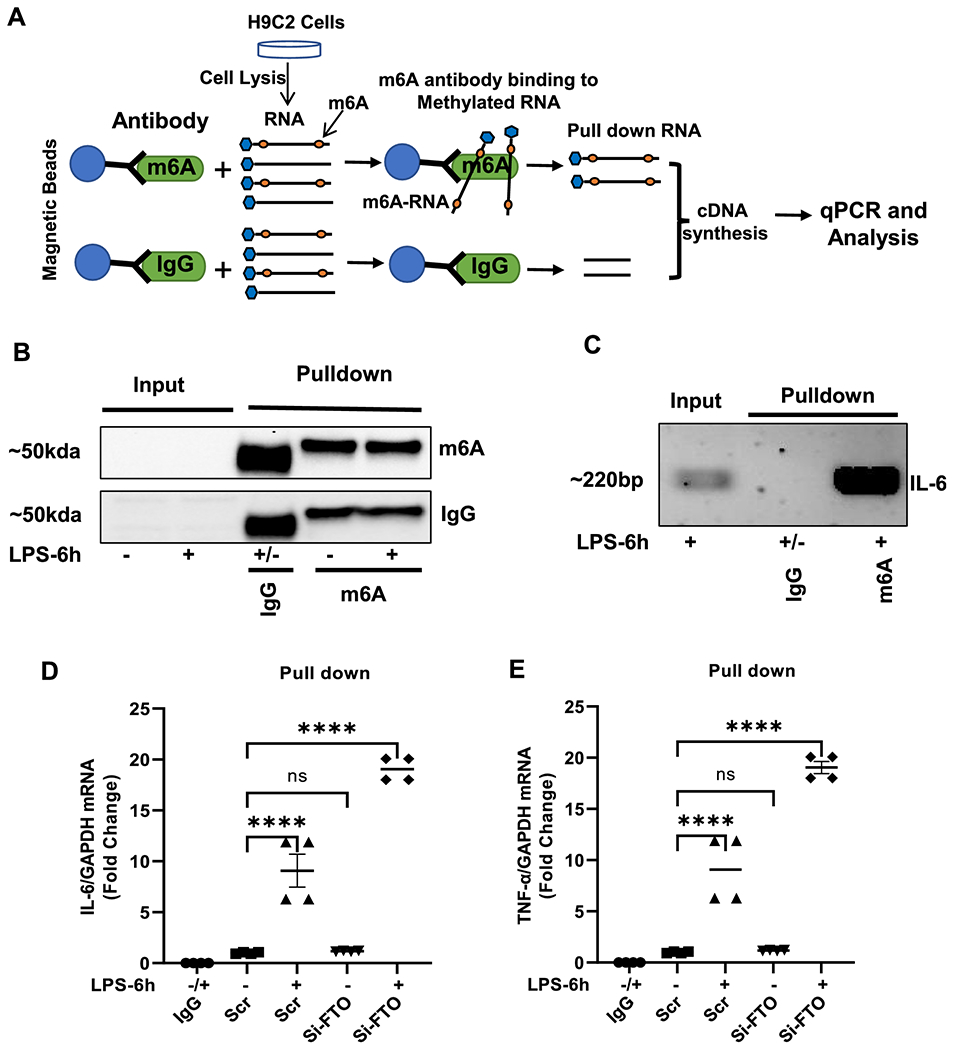
MeRIP assay in H9c2 cells after knocking down of FTO. (A) Schematic representation of MeRIP assay protocol to pull down methylated RNA using m6A and IgG (control) antibodies. (B) Quality control immunoblot for IgG and m6A antibody after MeRIP. (C) Semi quantitative PCR of IL-6 after MeRIP. (D,E) Relative expression of IL-6 and TNF-α mRNA in FTO knockdown and Scr (Scramble) control cells treated with and without LPS after MeRIP. All data are represented as means SEMs. Data were analyzed using one-way ANOVA following Bartlett’s statistic (corrected) method to compare each group. ****P<0.0001

The decrease in FTO expression was accompanied by an elevation in IL-6 and TNF-α in the myocardium of endotoxemic mice, as well as in the LPS-treated H9c2 cells. To address if the upregulation of these cytokines was due to FTO-dependent m6A-RNA modification, we performed MeRIP assay (shown in Fig. 7A). Total RNA was extracted from FTO-knockdown and scramble-control H9c2 cells (with or without LPS stimulation for 6 hours). M6A-methylated mRNA was pulled-down using anti-m6A or IgG antibodies and the yield is shown in Supplementary Figure S4A. Quality check for IgG and M6A samples after pull-down is shown in immunoblot (Fig. 7B). Furthermore, IL-6 mRNA was evaluated by semi-quantitative PCR in the input and MeRIP-pulldown RNA samples (Fig. 7C). Next, qPCR analyses showed amplification of IL-6 and TNF-α transcripts in the m6A-RNA pulldown samples but not in the IgG control pulldown samples suggesting an increase in m6A methylation of transcripts after LPS stimulation (Fig. 7D and 7E). Interestingly, m6A methylation of IL-6 and TNF-α mRNAs was markedly enhanced in FTO-knockdown cells when compared to scrambled control cells (Fig. 7D and 7E). These findings suggest that downregulation of FTO during endotoxemia causes hypermethylation of IL-6 and TNF-α transcripts. Also, IL-6 and TNF-α mRNA expression in the input RNA showed similar trend after LPS stimulation or silencing of FTO (Supplementary Fig. S4B and S4C).

## Discussion

Cardiac pathology and dysfunction, characterized by ventricular dilatation and compromised ventricular contractility, is an important component of multiorgan failure caused by severe sepsis [1, 2, 21]. Proinflammatory cytokine production within the myocardium has been attributed to LV remodeling and impairment in cardiac function. Elevated levels of these cytokines could interfere with nitric oxide signaling, calcium homeostasis, and β-adrenergic signaling [22, 23]. LPS-induced endotoxemia causes rapid production of proinflammatory cytokines in the myocardium that results in cardiac inflammation, cardiomyocyte apoptosis, and endothelial dysfunction [2, 6, 23]. Despite the knowledge of sepsis-associated cardiac complications, the underlying mechanisms of cardiac inflammation and dysfunction are still elusive.

N6-methyladenosine (m6A) is one of the most common RNA modifications in eukaryotic mRNA [8, 15]. m6A-RNA modification alters several biological processes by regulating RNA splicing, transport, stability, and cellular localization [9, 10, 19]. Although m6A modifications are studied in various pathophysiological processes, their influence on endotoxemia-mediated cardiac inflammation is not well characterized. In the present study, we investigated the influence of endotoxemia on status of cardiac m6A methylation and associated effects on myocardial inflammation and function. We observed a significant increase in global m6A-RNA methylation in the cardiac tissues of LPS-induced endotoxemic mice as well as in LPS-treated H9c2 cardiomyoblasts. The change in m6A methylation status was associated with increased expression of proinflammatory cytokines.

m6A modification is a dynamic process, which is tightly regulated by RNA methyltransferases (writers, METTL3, METTL14, and WTAP), demethylases (erasers, FTO and ALKBH), and m6A-binding proteins (readers, YTH domain family members) [9, 24]. Alterations in these regulators have been reported in cardiac pathophysiology, including cardiac remodeling and heart failure [11, 14, 19, 25]. Berulava *et al.* has shown that m6A-RNA methylation was significantly altered during the progression of heart failure [15]. FTO and ALKBH5 are the only two erasers known, and both belong to the ALKB family of dioxygenases [9]. FTO and ALKBH5 oxidatively remove m6A methylated groups from mRNA [9, 13, 15, 25]. Recently, downregulation of FTO was reported in human and mouse failing hearts [13, 14]. FTO-dependent m6A methylation in the failing hearts led to compromised cardiomyocyte contractility, cardiac dysfunction, and remodeling [14]. Overexpression of FTO inhibited cardiac fibrosis, enhanced angiogenesis in the mice with myocardial infarction, and improved heart function by regulating calcium handling [13–15]. However, whether FTO is involved in the endotoxemia-associated m6A modification of myocardial RNAs is still unknown. Interestingly, in the current study, we observed a significant downregulation of FTO in the myocardium of LPS-administered mice, as well as in LPS-stimulated cardiomyoblast cells. Downregulation of FTO in response to LPS could be due to changes in FTO mRNA expression at transcriptional, post transcriptional levels or due to changes in FTO protein stability via proteasomal system. However, these mechanisms need to be further investigated. As discussed earlier, the downregulation of FTO was associated with increased m6A methylation. Furthermore, LPS treatment also triggered the upregulation of proinflammatory cytokines, such as IL-6 and TNF-α, in the mice hearts as well as in H9c2 cardiomyocytes. To confirm that the increase in expression of inflammatory markers was FTO-dependent, we silenced FTO in H9c2 cells using rat-specific FTO-siRNA. Knockdown of FTO in H9c2 cells resulted in significant upregulation of inflammatory cytokines IL-6, TNF-α and IL-10. In addition, knockdown of FTO also elevated the transcript levels of IL-6, TNF-α and IL10 without LPS stimulation; and these effects were amplified in response to LPS treatment, thus confirming that FTO-induced m6A methylation is involved in LPS-induced inflammation in cardiomyocytes. We also observed basal m6A methylation in untreated samples and anti-inflammatory gene IL-10 suggesting that m6A methylation is required to maintain cardiac homeostasis [11].

**Figure 8.**
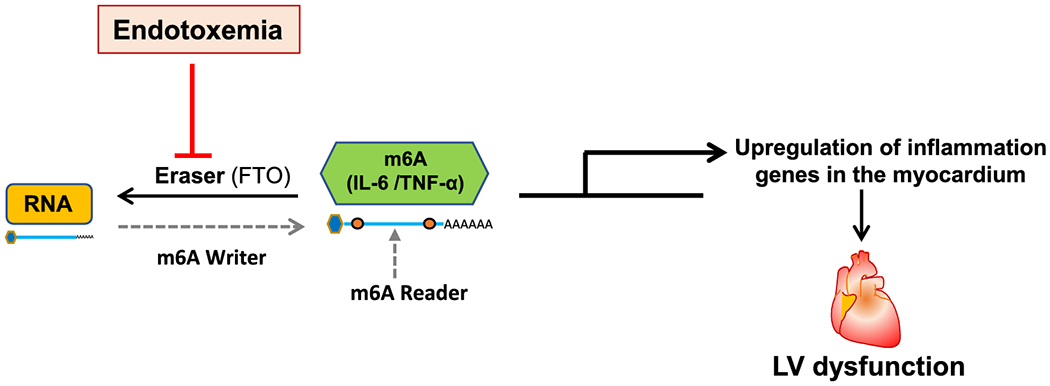
Graphical representation. Endotoxemia downregulates FTO expression in the myocardium, which results in hypermethylation of IL-6 and TNF-α transcripts and therefore increased inflammatory response in the myocardium leading to LV dysfunction.

An earlier study showed that FTO-dependent hypermethylation of SERCA2a and RY2R mRNAs altered calcium signaling and cardiac contractility in failing hearts [13, 14]. To investigate whether endotoxemia-induced FTO downregulation is associated with hypermethylation of inflammatory cytokines, we performed a MeRIP assay, and analyzed the expression of IL-6 and TNF-α by qPCR in FTO-silenced H9c2 cells with or without LPS stimulation. Interestingly, we observed that IL-6 and TNF-α mRNAs were hypermethylated in FTO-knockdown cells after LPS stimulation. These observations suggest that LPS-induced myocardial inflammatory surge is linked to hypermethylation of inflammatory cytokines in an FTO-dependent manner. Also, it is possible that hypermethylation of IL-6 and TNF-α may affect the regulation of RNA splicing, transport, stability, and cellular localization by regulating interaction of RNA binding proteins or miRNA [26]. The specific mechanism of action of m6A methylation in cardiac function still needs further investigation. We hypothesize that in normal condition, to maintain cellular homeostasis, appropriate level of m6A methylation is continuously regulated by expression of writers and erasers. However, altered expression of writers or erasers could change m6A methylation patterns. In endotoxemia, downregulation of erasers (*e.g*., FTO) leads to enhanced m6A methylation of inflammatory cytokines in the myocardium. These changes drive aberrant gene expression and signaling mechanisms leading to cardiac dysfunction (graphical representation of our working model is shown in Fig. 8).

In conclusion, our data demonstrates that endotoxemia alters the expression of m6A demethylase FTO and subsequent m6A methylation in the cardiac tissue. These changes lead to hypermethylation of proinflammatory cytokines, which might increase the expression of target transcripts, potentially through increased RNA stability and protein translation. Taken together, our study also raises the possibility that FTO could be a potential target to attenuate cardiac inflammation and dysfunction during endotoxemia or sepsis. Further investigations are warranted for a comprehensive understanding of the m6A writers and erasers in the context of sepsis.

## Supplementary Material

1751494_Sup_info

## Data Availability

The datasets used or analyzed during the current study are available from the corresponding author on reasonable request.
