# Supplementary material for: Increased m6A-RNA methylation and FTO suppression is associated with myocardial inflammation and dysfunction during endotoxemia in mice
Source: Mol Cell Biochem. Author manuscript; Available in PMC 2023 Jan 1. (PMC8758538; doi:10.1007/s11010-021-04267-2)
Supplement: 1751494_Sup_info [file NIHMS1751494-supplement-1751494_Sup_info.pptx]

## Slide 1
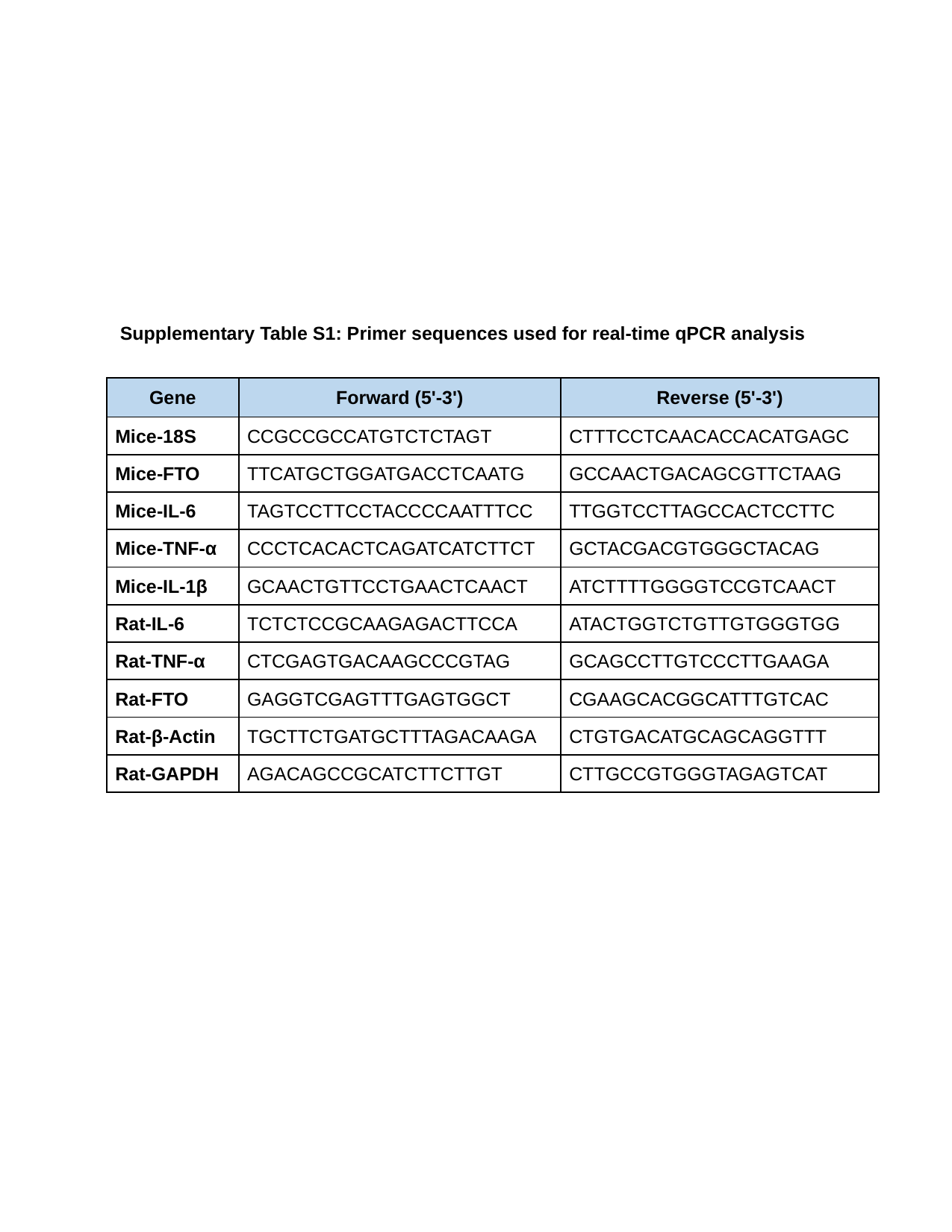

Supplementary Table S1: Primer sequences used for real-time qPCR analysis
| Gene | Forward (5'-3') | Reverse (5'-3') |
| --- | --- | --- |
| Mice-18S | CCGCCGCCATGTCTCTAGT | CTTTCCTCAACACCACATGAGC |
| Mice-FTO | TTCATGCTGGATGACCTCAATG | GCCAACTGACAGCGTTCTAAG |
| Mice-IL-6 | TAGTCCTTCCTACCCCAATTTCC | TTGGTCCTTAGCCACTCCTTC |
| Mice-TNF-α | CCCTCACACTCAGATCATCTTCT | GCTACGACGTGGGCTACAG |
| Mice-IL-1β | GCAACTGTTCCTGAACTCAACT | ATCTTTTGGGGTCCGTCAACT |
| Rat-IL-6 | TCTCTCCGCAAGAGACTTCCA | ATACTGGTCTGTTGTGGGTGG |
| Rat-TNF-α | CTCGAGTGACAAGCCCGTAG | GCAGCCTTGTCCCTTGAAGA |
| Rat-FTO | GAGGTCGAGTTTGAGTGGCT | CGAAGCACGGCATTTGTCAC |
| Rat-β-Actin | TGCTTCTGATGCTTTAGACAAGA | CTGTGACATGCAGCAGGTTT |
| Rat-GAPDH | AGACAGCCGCATCTTCTTGT | CTTGCCGTGGGTAGAGTCAT |

## Slide 2
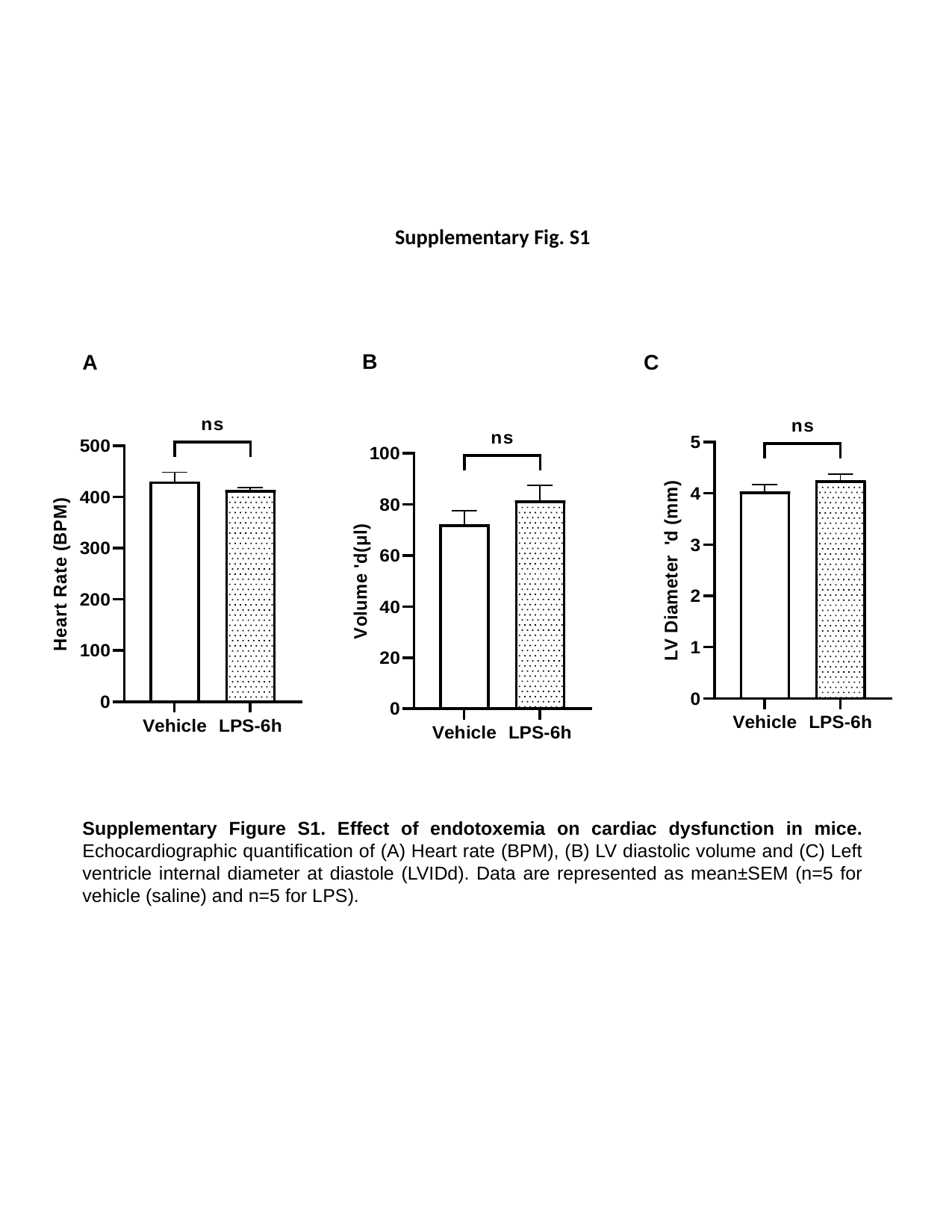

Supplementary Fig. S1
B
A
C
Supplementary Figure S1. Effect of endotoxemia on cardiac dysfunction in mice. Echocardiographic quantification of (A) Heart rate (BPM), (B) LV diastolic volume and (C) Left ventricle internal diameter at diastole (LVIDd). Data are represented as mean±SEM (n=5 for vehicle (saline) and n=5 for LPS).

## Slide 3
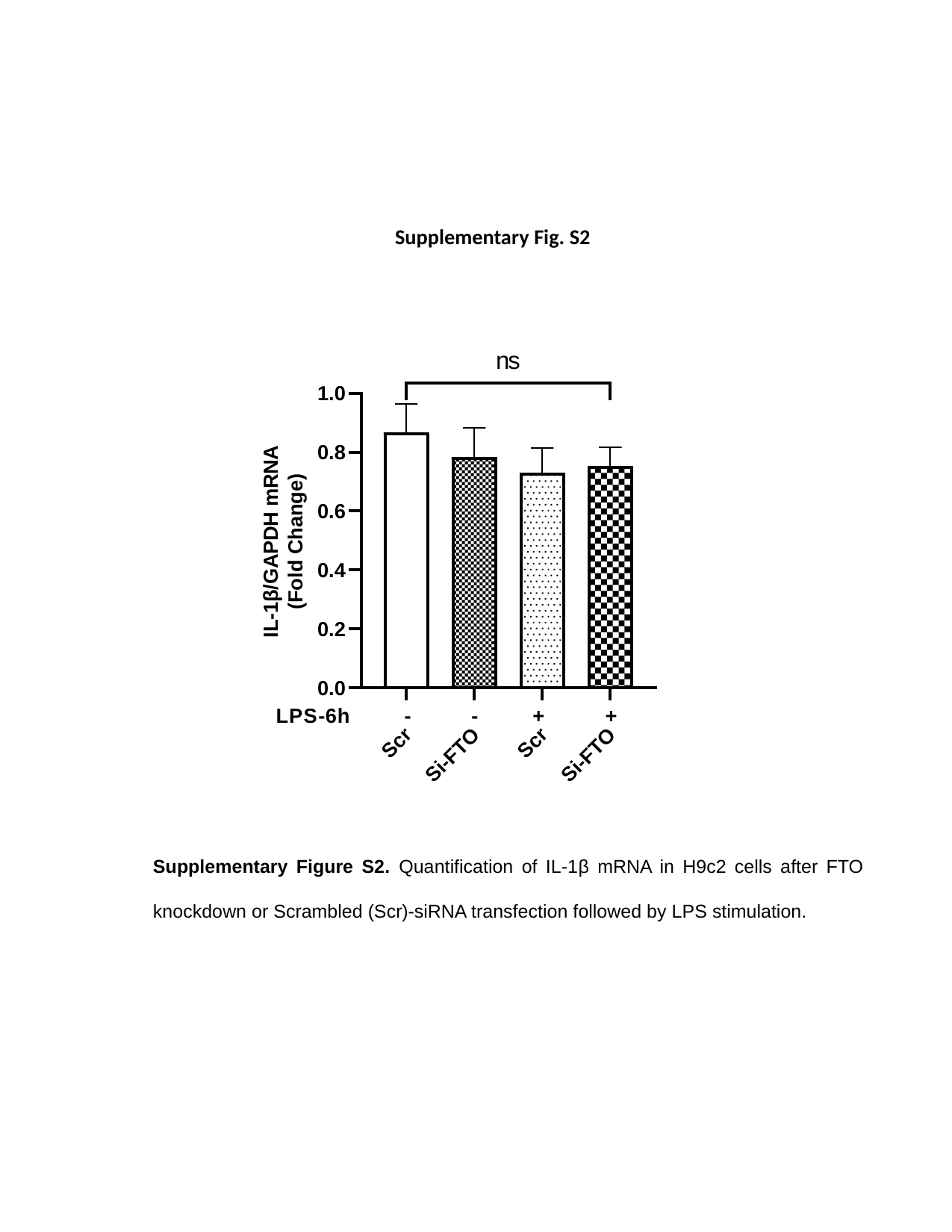

Supplementary Fig. S2
Supplementary Figure S2. Quantification of IL-1β mRNA in H9c2 cells after FTO knockdown or Scrambled (Scr)-siRNA transfection followed by LPS stimulation.

## Slide 4
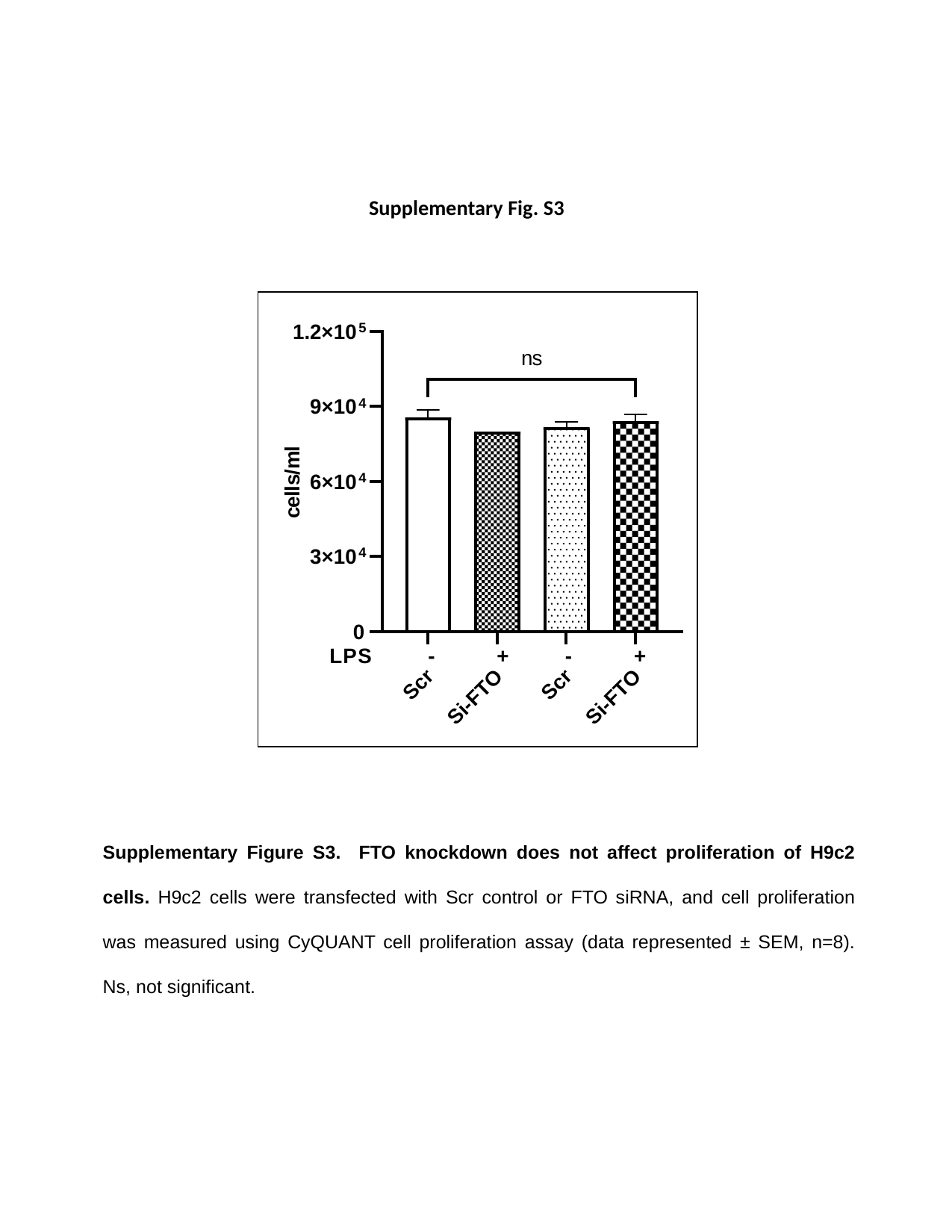

Supplementary Fig. S3
Supplementary Figure S3. FTO knockdown does not affect proliferation of H9c2 cells. H9c2 cells were transfected with Scr control or FTO siRNA, and cell proliferation was measured using CyQUANT cell proliferation assay (data represented ± SEM, n=8). Ns, not significant.

## Slide 5
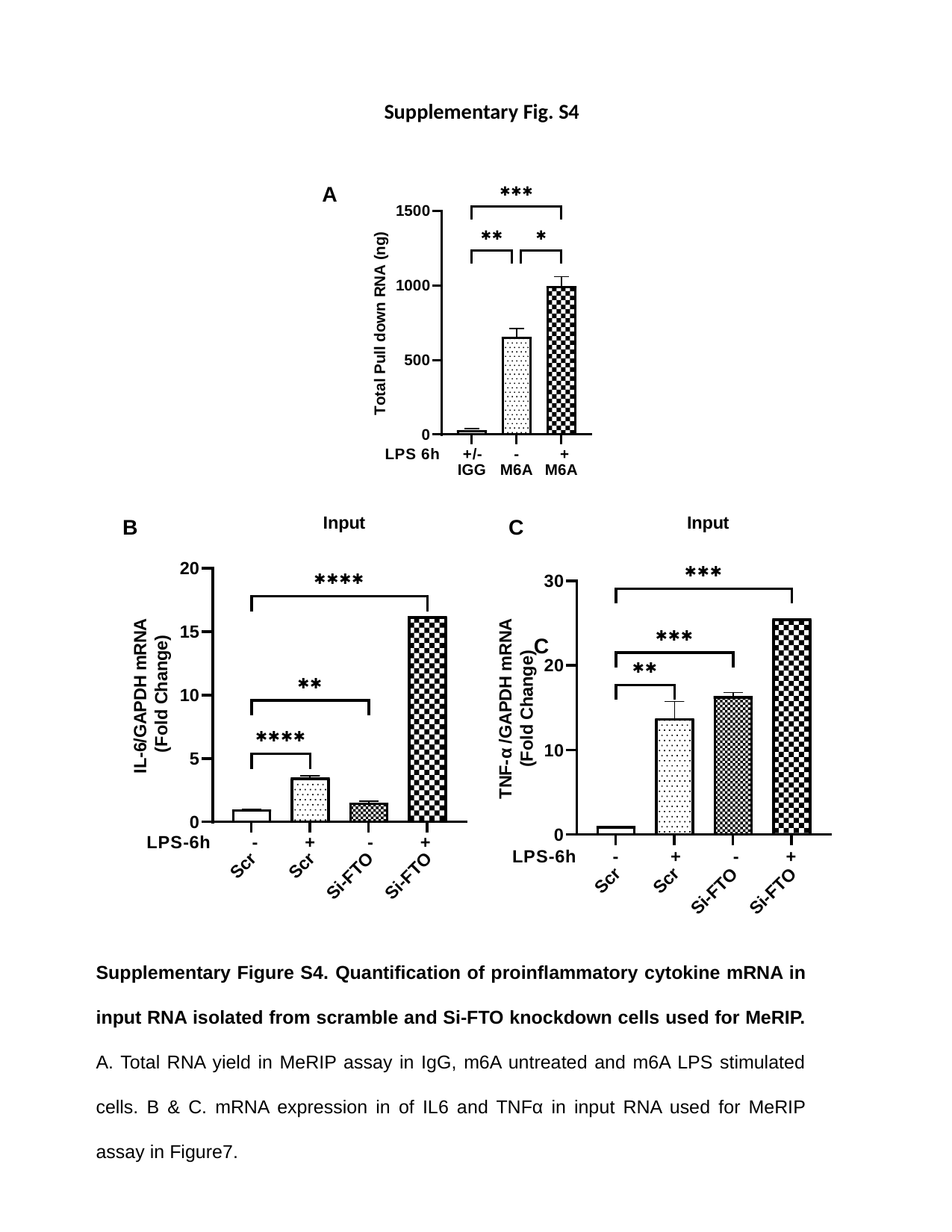

Supplementary Fig. S4
A
B
C
C
Supplementary Figure S4. Quantification of proinflammatory cytokine mRNA in input RNA isolated from scramble and Si-FTO knockdown cells used for MeRIP. A. Total RNA yield in MeRIP assay in IgG, m6A untreated and m6A LPS stimulated cells. B & C. mRNA expression in of IL6 and TNFα in input RNA used for MeRIP assay in Figure7.
